# Variational Bayesian Based Adaptive Shifted Rayleigh Filter for Bearings-Only Tracking in Clutters

**DOI:** 10.3390/s19071512

**Published:** 2019-03-28

**Authors:** Jing Hou, Yan Yang, Tian Gao

**Affiliations:** School of Electronic and Information, Northwestern Polytechnical University, Xi’an 710072, China; jhou0825@nwpu.edu.cn (J.H.); tiangao@nwpu.edu.cn (T.G.)

**Keywords:** bearings-only tracking, clutter, variational Bayesian, Shifted Rayleigh Filter

## Abstract

This paper considers bearings-only target tracking in clutters with uncertain clutter probability. The traditional shifted Rayleigh filter (SRF), which assumes known clutter probability, may have degraded performance in challenging scenarios. To improve the tracking performance, a variational Bayesian-based adaptive shifted Rayleigh filter (VB-SRF) is proposed in this paper. The target state and the clutter probability are jointly estimated to account for the uncertainty in clutter probability. Performance of the proposed filter is evaluated by comparing with SRF and the probability data association (PDA)-based filters in two scenarios. Simulation results show that the proposed VB-SRF algorithm outperforms the traditional SRF and PDA-based filters especially in complex adverse scenarios in terms of track continuity, track accuracy and robustness with a little higher computation complexity.

## 1. Introduction

Bearings-only target tracking is to estimate the current position and velocity of a target using only the noise-corrupted bearing measurements from one or multiple observer platforms. It is an important tracking problem that arises in both military and civilian applications, such as underwater sonar tracking, bistatic radar, air traffic control and computer vision [1].

Because of the intrinsic nonlinearities in the measurement models, it is difficult to acquire an optimal solution of this problem. Several suboptimal algorithms have been developed for bearings-only tracking in the literature. The extended Kalman filter (EKF) [2] in the Cartesian coordinate system is an early attempt. However, it is easy to diverge. To improve the stability of the EKF, the bearings-only tracking problem was formulated in modified polar coordinates, resulting in the modified polar coordinate EKF (MPEKF) [3]. However, it requires good initialization to guarantee convergence. The well-known pseudo-linear estimator (PLE) [4] was also developed to solve the bearings-only tracking problem. However, it gives a biased estimate at long ranges. In recent years, some bias compensation techniques were developed to improve the performance of PLE [5,6,7]. In addition, more sophisticated nonlinear Kalman filtering algorithms, such as unscented Kalman filter (UKF) [8,9], cubature Kalman filter (CKF) [1] and particle filter (PF) [10,11] were applied for bearings-only target tracking. PF can provide good performance but at the price of heavy computation load. Noteworthily, Clark et al. [12,13] proposed a novel shifted Rayleigh filter (SRF) for bearings-only tracking, which is still based on the approximation of conditional expectations but with novel feature which is performing a calculation to exploit the essential structure of the nonlinearities in a new way. It is shown to exhibit similar performance to the PF in certain challenging scenarios with much lower computational complexity [14].

However, these algorithms do not consider the impact of clutter which makes the bearings-only tracking problem more intractable. Apparently, the classical treatment of clutter in target tracking problem can be extended to the bearings-only tracking problem. For example, Reference [15] integrated the maximum entropy fuzzy probabilistic data association (MEFPDA) with the square-root cubature Kalman filter (SCKF) to deal with the clutter in bearings-only tracking. Clark et al. also included the effect of clutter measurements into the SRF algorithm in [12]. However, in the classical algorithms, the clutter probability is usually assumed known and constant, which maybe time-varying or hard to determine in advance especially in adverse scenarios. Use of incorrect value of the clutter probability may lead to track accuracy degradation even track loss. A straight-forward idea is to account for the unknown clutter probability in the process of estimation of the state. That is, we need to solve the problem of bearings-only target tracking with uncertain parameter of clutter probability.

As we know, the Bayesian approach is the most general approach of solving the problem with uncertain parameters. However, it is not trivial to get the analytical solution for most Bayesian approaches due to complex nonlinear probability density function or high dimension of integration. Recently, the variational Bayesian (VB)-based adaptive filters [16,17,18] have drawn extensive attention, which utilize a new simpler, analytically tractable distribution to approximate the true posterior distribution so that avoiding direct calculations of complex integrals. Its adaptive strategy has a strong ability of tracking time-varying parameters. Therefore, we adopt the VB method to jointly estimate the target state and the clutter probability within the framework of SRF in this paper for bearings-only tracking in clutters. By establishing a conjugate exponential model for clutter probability and data association indicator, the proposed filter is derived using the iterative filtering framework. The tracking performance of the proposed VB-SRF is evaluated by comparing with SRF, PDA-SCKF [15] and MEFPDA-SCKF [15] via two simulation examples. It shows that the proposed filter outperforms the traditional SRF and two PDA-SCKF-based filters in complex mismatched scenarios in terms of track continuity and track accuracy but at the cost of higher computation complexity.

The remainder of the paper is organized as follows. Section 2 gives the problem formulation. In Section 3, the variational Bayesian filtering is described. Section 4 derives the VB-based adaptive SRF. Section 5 provides simulation results and performance evaluation of the proposed approach, followed by the conclusions in Section 6.

## 2. The Shifted Rayleigh Filter Algorithm

### 2.1. The Bearing Model

Considering the shifted Rayleigh filter (SRF) for bearings-only tracking in $R^{2}$, the state equation and the measurement equation are described as [13]:

(1) $$\begin{array}{cc} & \;\;\;x_{k}\;=F_{k-1}x_{k-1}+u_{k-1}^{s}+v_{k-1} \end{array}$$

(2) $$\begin{array}{c} \begin{array}{cc} y_{k} & =H_{k}x_{k}+u_{k}^{m}+w_{k}\\ b_{k} & =\Pi (y_{k}) \end{array} \end{array}$$

where, $x_{k}$ is the state vector which describes the position and velocity of the target; $b_{k}$ is the noisy bearing measurement. Π denotes the projection of the plane onto the unit circle. That is taking a 2-vector $y_{k}$ into its normalized form $y_{k}/\left|\right|y_{k}\left|\right|$. Then $b_{k}={\left(sin\theta_{k},cos\theta_{k}\right)}^{T}$, where $\theta_{k}$ is the bearing of the target position relative to the sensor platform. $F_{k-1}$ and $H_{k}$ are the state transition matrix and measurement matrix, respectively. $u_{k-1}^{s}$ and $u_{k}^{m}$ are the inputs to the system to increase the versatility of the model, for example, to reflect known perturbations to the dynamics and changes in sensor location; $v_{k-1}$ is the Gaussian process noise with zero mean and covariance $Q_{v}$, and $w_{k}$ is the Gaussian measurement noise with zero mean and covariance $Q_{w}$ and independent of $v_{k}$.

The unusual point in the measurement model is that the noise $w_{k}$ is modelled as additive noise present in an “augmented” measurement, $y_{k}$, of the Cartesian coordinates of target relative to the sensor platform, which is projected onto the plane to generate the actual bearing measurement $b_{k}$. It is different with the traditional “angle plus white noise” model, expressed as

(3) $$\theta_{k}=arctan\left(d_{1}/d_{2}\right)+\epsilon_{k}$$

where, $d_{1},d_{2}$ are the components of the displacement vector $d_{k}=H_{k}x_{k}+u_{k}^{m}$. $\epsilon_{k}$ is the sensor noise with Gaussian distribution $N(0,\sigma^{2})$ and independent of the displacement vector $d_{k}$.

Actually, as explained in [13], the shifted Rayleigh bearing model (2) can be related with the traditional model (3) by making a variant on the shifted Rayleigh noise model

(4) $$\begin{array}{cc} y_{k}^{\prime } & =d_{k}+\left|\right|d_{k}\left|\right|e^{\prime }\\ b_{k}^{\prime } & =\Pi (y_{k}^{\prime }) \end{array}$$

where, $e^{\prime }$ is an $N(0,\sigma^{2}I_{2\times 2})$ distributed random variable. The only difference with model (2) is the noise term $w_{k}$ used to construct the augmented measurement $y_{k}$ is replaced by $\left|\right|d_{k}\left|\right|e^{\prime }$. $\left|\right|d_{k}\left|\right|e^{\prime }$ differs from $w_{k}$, but has identical first and second moments, and is uncorrelated with $d_{k}$.

The angular $\theta_{k}^{\prime }$ of the modified vector bearing $b_{k}^{\prime }$ can be represented as

(5) $$\theta_{k}^{\prime }=arctan\left(d_{1}/d_{2}\right)+\epsilon_{k}^{\prime }$$

where $\epsilon_{k}^{\prime }$ is a zero mean random variable, restricted to [−π,π], independent of $d_{k}$, with density $\alpha_{\sigma }\left(\cdot \right)$:

(6) $$\alpha_{\sigma }\left(\theta \right)=\frac{e^{-1/2\sigma^{2}}}{2\pi }\left(1+\sqrt{2\pi }\frac{cos\theta }{\sigma }F_{normal}\left(\frac{cos\theta }{\sigma }\right)e^{1/2{\left(cos\theta /\sigma \right)}^{2}}\right)$$

where, $F_{normal}\left(\cdot \right)$ is the cumulative distribution function of a standard N(0,1) variable.

Note that $\theta^{\prime }$ given by (5), is very close to the bearing θ in the standard model (3). The only difference is that the densities of the noise terms used in their construction are $\alpha_{\sigma }\left(\theta \right)$ and the normal $N(0,\sigma^{2})$ density, respectively. Reference [13] plots the two densities for $\sigma^{2}\leq 1$. It shows the two density functions are virtually indistinguishable.

Given the bearing model, the SRF is to calculate the estimates of the conditional mean and covariance of the target state $x_{k}$, given measurements up to time *k*, $b_{1:k}$. That is,

(7) $${\hat{x}}_{k|k}=E\left[x_{k}|b_{1:k}\right],\;P_{k|k}=cov\left[x_{k}|b_{1:k}\right]$$

The formulas for the SRF algorithm can be seen in [13].

### 2.2. The Treatment of Clutter

Accounting for the effects of clutter on the bearing measurements, we represent a cluttered bearing measurement as

(8) $$z_{k}=\left(1-r_{k}\right)\theta_{k}+r_{k}U_{k}$$

where $U_{k}$ is the bearing measurement of the clutter, which is assumed to be an uniform random variable on [−π,π]; $\theta_{k}$ is the bearing measurement of the actual target from the sensor, $r_{k}$ is defined as data association indicator at time *k*.

(9) $$\begin{array}{c} r_{k}=\left\{\begin{array}{cc} 1 & \mathrm{if}\mathrm{the}\mathrm{measurement}\mathrm{is}\mathrm{from}\mathrm{clutter}\\ 0 & \mathrm{if}\mathrm{the}\mathrm{measurement}\mathrm{is}\mathrm{from}\mathrm{target} \end{array}\right. \end{array}$$

The prior of $r_{k}$ is assumed independent of the previous data associations and can be described as

(10) $$\begin{array}{c} p\left(r_{k}\right)=\left\{\begin{array}{cc} \xi & \mathrm{if}\;r_{k}=1\\ 1-\xi & \mathrm{if}\;r_{k}=0 \end{array}\right. \end{array}$$

Then the likelihood of the measurement is

(11) $$\begin{array}{c} p\left(z_{k}|x_{k},r_{k}\right)=\left\{\begin{array}{cc} 1/2\pi & if\;r_{k}=1\\ f(\theta_{k}|x_{k}) & if\;r_{k}=0 \end{array}\right. \end{array}$$

It can be represented in two forms:

(12)p(zk|xk,rk)=12πrk+f(θk|xk)(1−rk)(13)=(12π)rkf(θk|xk)1−rk

Note that the two forms are used for different purpose in the later section. $f(\theta_{k}|x_{k})$ is the likelihood of the target bearing measurement, whose expression is given in Appendix A.

Here, we need to explain the reason for using this representation (8) of the cluttered measurement. The information carried in (8) is identical with the projection measurement $z_{k}^{b}=\left[sinz_{k},cosz_{k}\right]$. So the mean and covariance of the target state $x_{k}$ conditioned on cluttered measurement $z_{k}^{b}$, which are the aims needs to be achieved in the SRF, are equivalent with these conditioned on $z_{k}$. Furthermore, this representation (8) is more convenient for calculation. Thus, $z_{k}$ instead of $z_{k}^{b}$ is adopted to represent the clutter measurement.

Based on the cluttered measurement model, given clutter probability ξ, the state estimate and covariance can be calculated as follows.

Suppose the density of $x_{k-1}$ given measurements up to k−1, $p_{k-1|k-1}\left(x_{k-1}\right)$, is normal with mean ${\bar{x}}_{k-1|k-1}$ and covariance ${\bar{P}}_{k-1|k-1}$. The posterior density of state $x_{k}$ conditioned on cluttered bearing measurements $z_{1:k}$ is given as

p(xk|z1:k)=p(xk|zk,xk−1∼pk−1|k−1(xk−1))(14)=qk(0)pk|k(xk)+qk(1)pk|k−1(xk)

where $q_{k}\left(i\right)=p\left(r_{k}=i|z_{k},z_{1:k-1}\right)$, $p_{k|k}\left(x_{k}\right)$ is the non-normal density of $x_{k}$ conditioned on $r_{k}=0$ and $z_{k}$, or, equivalently, on $\theta_{k}$, and $p_{k|k-1}\left(x_{k}\right)$ is the density of $x_{k}$ when there is no target measurement at time *k*. It is normal with mean ${\hat{x}}_{k|k-1}$ and covariance $P_{k|k-1}$. Thus, the state estimate and covariance at time *k* can be obtained as

x¯k|k=E[xk|zk,xk−1∼pk−1|k−1(xk−1)](15)=qk(0)x^k|k+qk(1)x^k|k−1

P¯k|k=cov[xk|zk,xk−1∼pk−1|k−1(xk−1)]=qk(0)(Pk|k+(x^k|k−x¯k|k)(x^k|k−x¯k|k)T)(16)+qk(1)(Pk|k−1+(x^k|k−1−x¯k|k)(x^k|k−1−x¯k|k)T)

where ${\hat{x}}_{k|k}$ and $P_{k|k}$ are the state estimate and its covariance based on the actual target measurement. ${\hat{x}}_{k|k-1}$ and $P_{k|k-1}$ are the predicted target state estimate and covariance. All of these can be obtained using the basic formulas of SRF.

The conditional densities $q_{k}\left(0\right)$ and $q_{k}\left(1\right)$ are given by the equations

(17)qk(0)=ck(1−ξ)fk(θk|z1:k−1)(18)qk(1)=ckξ2π

where $c_{k}$ is the normalizing constant and $f_{k}\left(\theta_{k}|z_{1:k-1}\right)$ is the density of the actual target bearing $\theta_{k}$ conditioned on measurements $z_{1:k-1}$. The expression is given in Appendix B.

However, in a complex environment, the probability of clutter is time-varying or hard to determine in advance. In this case, the probability ξ is unknown, so the above formulas are not applicable. In this paper, we resort the VB method to find the joint posterior density of $x_{k}$ and ξ so as to account for the uncertainty in clutter probability.

## 3. Variational Bayesian Filtering

In this section, we first review the conjugate exponential (CE) model, and then gives the variational Bayesian solution of the CE model.

### 3.1. Conjugate Exponential Model

Given measurements $z_{1:k-1}$, the posterior of the system state $p(x_{k-1}|z_{1:k-1})$ and the posterior of the parameter $p(r_{k-1}|z_{1:k-1})$, we assume the complete-data likelihood in the exponential family:

(19) $$p\left(x_{k},z_{k}|r_{k},z_{1:k-1}\right)=g\left(r_{k}\right)f\left(x_{k},z_{k}\right)e^{\phi {\left(r_{k}\right)}^{T}u\left(x_{k},z_{k}\right)}$$

where, $\phi (r_{k})$ is the vector of natural parameters $r_{k}$, *u* and *f* are known functions, and *g* is a normalization constant:

$$g{\left(r_{k}\right)}^{-1}=\int f\left(x_{k},z_{k}\right)e^{\phi {\left(r_{k}\right)}^{T}u\left(x_{k},z_{k}\right)}dx_{k}dz_{k}$$

The parameter prior is conjugate to the complete-data likelihood:

(20) $$p\left(r_{k}|\alpha_{k}^{-},\beta_{k}^{-}\right)=h\left(\alpha_{k}^{-},\beta_{k}^{-}\right)g{\left(r_{k}\right)}^{\beta_{k}^{-}}e^{\phi {\left(r_{k}\right)}^{T}\alpha_{k}^{-}}$$

where $\alpha_{k}^{-}$ and $\beta_{k}^{-}$ are hyperparameters of the prior, and *h* is a normalization constant. Note the prior $p(r_{k}|\alpha_{k}^{-},\beta_{k}^{-})$ is said to be conjugate to the likelihood $p(x_{k},z_{k}|r_{k})$ if and only if the posterior

$$p\left(r_{k}|\alpha_{k},\beta_{k}\right)\propto p\left(r_{k}|\alpha_{k}^{-},\beta_{k}^{-}\right)p\left(x_{k},z_{k}|r_{k}\right)$$

is of the same parametric form as the prior. Then we call models that satisfy Equations (19) and (20) conjugate-exponential.

### 3.2. VB Approximation Method

Applying Bayes’ rule, we have the joint posterior of $x_{k}$ and $r_{k}$ as

(21) $$p\left(x_{k},r_{k}|z_{1:k}\right)\propto p\left(x_{k},z_{k}|r_{k},z_{1:k}\right)p\left(r_{k}|\alpha_{k}^{-},\beta_{k}^{-}\right)$$

The analytic solution to (21) would be difficult to calculate. Here, we use the VB method to approximate the true posterior distribution with a product of tractable marginal posteriors [17].

(22) $$p\left(x_{k},r_{k}|z_{1:k}\right)\approx Q\left(x_{k},r_{k}\right)=Q_{x}\left(x_{k}\right)Q_{r}\left(r_{k}\right)$$

where $Q_{x}\left(x_{k}\right)$ and $Q_{r}\left(r_{k}\right)$ are unknown approximating marginal densities of $x_{k}$ and $r_{k}$.

The basic idea of VB approximation is to minimize the Kullback- Leibler (KL) divergence between the approximating posterior and the true posterior:

(23) $$\begin{array}{c} KL\left[Q_{x}\left(x_{k}\right)Q_{r}\left(r_{k}\right)\left|\right|p\left(x_{k},r_{k}|z_{1:k}\right)\right]=\int Q_{x}\left(x_{k}\right)Q_{r}\left(r_{k}\right)\times \log\left(\frac{Q_{x}\left(x_{k}\right)Q_{r}\left(r_{k}\right)}{p(x_{k},r_{k}|z_{1:k})}\right)dx_{k}dr_{k} \end{array}$$

Given the measurements $z_{1:k}$, we can minimize the KL divergence with respect to the probability densities $Q_{x}\left(x_{k}\right)$ and $Q_{r}\left(r_{k}\right)$ in turn, while keeping the other fixed. Then, the following equations can be given as:

(24) $$Q_{x}\left(x_{k}\right)\propto exp\left({\langle \ln p\left(x_{k},r_{k},z_{k}|z_{1:k-1}\right)\rangle }_{r_{k}}\right)$$

(25) $$Q_{r}\left(r_{k}\right)\propto exp\left({\langle \ln p\left(x_{k},r_{k},z_{k}|z_{1:k-1}\right)\rangle }_{x_{k}}\right)$$

where ${\langle \cdot \rangle }_{x_{k}}$ and ${\langle \cdot \rangle }_{r_{k}}$ denote the expectations with respect to $Q_{x}\left(x_{k}\right)$ and $Q_{r}\left(r_{k}\right)$, respectively. Obviously, it is not an explicit solution since the distribution of each parameter is dependent on the other and neither distributions is known. The mechanism of VB method is to firstly give the initial values of the parameters and then use expectation-maximum (EM) algorithm to iteratively calculate $Q_{x}\left(x_{k}\right)$ and $Q_{r}\left(r_{k}\right)$ until convergence. For the above CE models, $Q_{x}\left(x_{k}\right)$ and $Q_{r}\left(r_{k}\right)$ can be obtained from the following procedure [19]:

(1) The VB expectation step yields:
  (26) $$Q_{x}\left(x_{k}\right)\propto f\left(x_{k},z_{k}\right)e^{{\langle \phi (r_{k})\rangle }_{r_{k}}^{T}u\left(x_{k},z_{k}\right)}=p\left(x_{k}|z_{k},{\langle \phi (r_{k})\rangle }_{r_{k}}\right)$$
(2) The VB maximization step yields that $Q_{r}\left(r_{k}\right)$ is conjugate and of the form
  (27) $$Q_{r}\left(r_{k}\right)=h(\alpha_{k},\beta_{k}^{-}g{\left(r_{k}\right)}^{\beta_{k}}e^{\phi {\left(r_{k}\right)}^{T}\alpha_{k}}$$
  where, $\alpha_{k}$ and $\beta_{k}$ are the hyper-parameters, and
  (28)αk=αk−+〈u(xk,zk)〉xk(29)βk=βk−+n
  where *n* is the dimension of the measurement.

## 4. VB Based Adaptive Shifted Rayleigh Filter with Unknown Clutter Probability

Considering the system model (1) and the measurement model (8) described in Section 2, we adopt the VB method within the SRF framework to get the joint estimation of the target state and the clutter probability.

The core is to determine the posterior approximation $Q(x_{k},r_{k},\xi )$. Assume factorization $Q\left(x_{k},r_{k},\xi \right)\approx Q_{x}\left(x_{k}\right)Q\left(r_{k},\xi \right)$, we can obtain $Q_{x}\left(x_{k}\right)$, $Q_{r}\left(r_{k}\right)$ and $Q_{\xi }\left(\xi_{k}\right)$ at each time *k* through the following procedure.

(1) Optimization of $Q_{x}\left(x_{k}\right)$ for fixed $Q(r_{k},\xi )$.

First, by using the first form of the measurement likelihood (12), the complete-data likelihood is presented as

p(xk,zk|rk,z1:k−1)=p(zk|xk,rk)p(xk|rk,z1:k−1)(30)=[12πrk+f(θk|xk)(1−rk)]N(xk;x^k|k−1,Pk|k−1)

Then according to (24), we can get

Qx(xk)∝exp{〈lnp(zk,xk|rk,z1:k−1)〉rk,ξ}=[12π〈rk〉rk+f(θk|xk)(1−〈rk〉rk)]N(xk;x^k|k−1,Pk|k−1)(31)=12πN(xk;x^k|k−1,Pk|k−1)〈rk〉rk+f(θk|xk)N(xk;x^k|k−1,Pk|k−1)(1−〈rk〉rk)(32)≈12πN(xk;x^k|k−1,Pk|k−1)〈rk〉rk+N(xk;x^k|k,Pk|k)f(θk|z1:k−1)(1−〈rk〉rk)

The approximation sign in (32) is because the following:

f(θk|xk)N(xk;x^k|k−1,Pk|k−1)=p(θk|xk,z1:k−1)p(xk|z1:k−1)=p(xk|θk,z1:k−1)p(θk|z1:k−1)(33)≈N(xk;x^k|k,Pk|k)f(θk|z1:k−1)

where $f(\theta_{k}|z_{1:k-1})$ is derived in Appendix B.

Comparing (32) with the posterior density (14) of SRF, the difference lies in the weights. Except for a normalization constant, the clutter probability ξ used before in (14) has been replaced by ${\langle r_{k}\rangle }_{r_{k}}$ in (32), which is updated online.

(2) Optimization of $Q(r_{k},\xi )$ for fixed $Q_{x}\left(x_{k}\right)$.

We use the VB method again by factorizing $Q\left(r_{k},\xi \right)\approx Q_{r}\left(r_{k}\right)Q_{\xi }\left(\xi \right)$. Assume the conjugate prior of $r_{k}$ is binomial distributed with parameter ξ. That is,

(34) $$\begin{array}{cc} p(r_{k}|\xi ) & =\xi^{r_{k}}{\left(1-\xi \right)}^{1-r_{k}} \end{array}$$

and p(ξ) follows beta distribution with parameters $\alpha_{1}$ and $\alpha_{2}$.

(35) $$p\left(\xi ;\alpha_{1},\alpha_{2}\right)=\frac{1}{B(\alpha_{1},\alpha_{2})}\xi^{\alpha_{1}-1}{\left(1-\xi \right)}^{\alpha_{2}-1}$$

where $B\left(\alpha_{1},\alpha_{2}\right)=\Gamma \left(\alpha_{1}\right)\Gamma \left(\alpha_{2}\right)/\Gamma \left(\alpha_{1}+\alpha_{2}\right)$.

To have the form of (19), the complete-data likelihood $p(x_{k},z_{k}|r_{k},z_{1:k-1})$ is re-derived using the second form (13) of $p(z_{k}|x_{k},r_{k})$ as

p(xk,zk|rk,z1:k−1)=(1/2π)rk[f(θk|xk)]1−rkN(xk;x^k|k−1,Pk|k−1)(36)=f(θk|xk)N(xk;x^k|k−1,Pk|k−1)exp{rk[ln(1/2π)−ln[f(θk|xk)]}

Rewriting the conjugate prior of $r_{k}$ in the form of (20), we can get

p(rk|ξ)=ξrk(1−ξ)1−rk(37)=(1−ξ)exp[rkln(ξ1−ξ)]

Then applying (27), $Q_{r}\left(r_{k}\right)$ can be obtained as

(38) $$Q_{r}\left(r_{k}\right)=\left(1-\eta_{k}\right)exp\left[r_{k}ln\left(\frac{\eta_{k}}{1-\eta_{k}}\right)\right]$$

where, $\eta_{k}$ is the hyper-parameter and updated as

(39) $$ln\left(\frac{\eta_{k}}{1-\eta_{k}}\right)={\langle ln\left(\frac{\xi }{1-\xi }\right)\rangle }_{\xi }+\langle {[ln\left(1/2\pi \right)-ln\left[f\left(\theta_{k}|x_{k}\right)\right]\rangle }_{x_{k}}$$

Likewise, rewriting the conjugate prior of ξ in the form of (20) , we can get

p(ξ)=1B(α1,α2)ξα1−1(1−ξ)α2−1=1B(α1,α2)(1−ξ)α1+α2−2(1−ξ)−(α1−1)ξα1−1(40)=1B(α1,α2)(1−ξ)α1+α2−2exp[(α1−1)ln(ξ1−ξ)]

Then applying (27), $Q_{\xi }\left(\xi \right)$ can be obtained as

(41) $$Q_{\xi }\left(\xi \right)=\frac{1}{B(\alpha_{1}^{\prime },\alpha_{2}^{\prime })}{\left(1-\xi \right)}^{\alpha_{1}^{\prime }+\alpha_{2}^{\prime }-2}exp\left[\left(\alpha_{1}^{\prime }-1\right)ln\left(\frac{\xi }{1-\xi }\right)\right]$$

with hyper-parameters

(42)α1′=α1+〈rk〉rk(43)α2′=α2+n−〈rk〉rk

where *n* is the dimension of the measurement.

According to the approximated posteriors of $Q_{r}\left(r_{k}\right)$ and $Q_{\xi }\left(\xi \right)$, ${\langle r_{k}\rangle }_{r_{k}}$ and ${\langle ln\left(\frac{\xi }{1-\xi }\right)\rangle }_{\xi }$ can be obtained as:

(44)〈rk〉rk=ηk(45)〈ln(ξ1−ξ)〉ξ=ψ(α1′)−ψ(α2′)

where ψ(·) is the digamma function.

Taking expectation and covariance on the posterior $Q_{x}\left(x_{k}\right)$, the conditional mean and covariance of the target state can then be obtained. We summarize the entire filtering procedure of the VB-based SRF (VB-SRF) in Algorithm 1.

**Algorithm 1** : VB-SRF.
**(1) Initialization**: ${\bar{x}}_{0|0}$, ${\bar{P}}_{0|0}$, $Q_{v}$, $Q_{w}$, $\eta_{0}$, $\alpha_{1,0}$, $\alpha_{2,0}$

**(2) Prediction:**



$\;\;\;{\hat{x}}_{k|k-1}=F_{k-1}{\bar{x}}_{k-1|k-1}+u_{k-1}^{s}$




$\;\;\;P_{k|k-1}=F_{k-1}{\bar{P}}_{k-1|k-1}F_{k-1}^{T}+Q_{v}$




$\;\;\;S_{k}=H_{k}P_{k|k-1}H_{k}^{T}+Q_{k}^{m}$




$\;\;\;\eta_{k|k-1}=\rho \eta_{k-1},\;\alpha_{1,k|k-1}=\rho \alpha_{1,k-1},\;\alpha_{2,k|k-1}=\rho \alpha_{2,k-1}$


where ρ is the scale factor and 0<ρ≤1.
**(3) Update:** the update of VB-SRF utilizes iterate filtering framework.
 **(3.a) First set**: ${\bar{x}}_{k|k}^{\left(0\right)}={\hat{x}}_{k|k-1}$, ${\bar{P}}_{k|k}^{\left(0\right)}=P_{k|k-1}$, $\eta_{k}^{\left(0\right)}=\eta_{k|k-1}$, $\alpha_{1,k}^{\left(0\right)}=\alpha_{1,k|k-1}$, $\alpha_{2,k}^{\left(0\right)}=\alpha_{2,k|k-1}$
 **(3.b) Calculate state estimation and its covariance using SRF when the measurement is from the target**:


$\;\;\;\;K_{k}=P_{k|k-1}H_{k}^{T}S_{k}^{-1}$




$\;\;\;\;\varepsilon_{k}={\left(b_{k}^{T}S_{k}^{-1}b_{k}\right)}^{-1/2}b_{k}^{T}S_{k}^{-1}\left(H_{k}{\hat{X}}_{k|k-1}+u_{k}^{m}\right)$




$\;\;\;\;\gamma_{k}={\left(b_{k}^{T}S_{k}^{-1}b_{k}\right)}^{-1/2}\rho_{n}\left(\varepsilon_{k}\right)$




$\;\;\;\;\delta_{k}={\left(b_{k}^{T}S_{k}^{-1}b_{k}\right)}^{-1/2}\left[2+\varepsilon_{k}\rho_{2}\left(\varepsilon_{k}\right)-\rho_{2}^{2}\varepsilon_{k}\right]$




$\;\;\;\;\rho_{2}\left(\varepsilon_{k}\right)=\frac{\varepsilon_{k}e^{-\varepsilon_{k}^{2}/2}+\sqrt{2\pi }\left(\varepsilon_{k}^{2}+1\right)F_{normal}\left(\varepsilon_{k}\right)}{e^{-\varepsilon_{k}^{2}/2}+\sqrt{2\pi }\left(\varepsilon_{k}\right)F_{normal}\left(\varepsilon_{k}\right)}$




$\;\;\;\;{\hat{x}}_{k|k}=\left(I-K_{k}H_{k}\right){\hat{x}}_{k|k-1}-K_{k}u_{k}^{m}+\gamma_{k}K_{k}b_{k}$




$\;\;\;\;P_{k|k}=\left(I-K_{k}H_{k}\right)P_{k|k-1}+\delta_{k}K_{k}b_{k}b_{k}^{T}K_{k}^{T}$


 **(3.c) For** j=1:N, iterate the following *N* (*N* denotes iterated times) steps:
• **Calculate the fused state estimation and its covariance**:


$\;\;\;\;{\bar{x}}_{k|k}^{\left(j\right)}=\frac{1}{2\pi c}\eta_{k}^{\left(j-1\right)}{\hat{x}}_{k|k-1}+\frac{1}{c}\left(1-\eta_{k}^{\left(j-1\right)}\right)f\left(\theta_{k}|z_{1:k-1}\right){\hat{x}}_{k|k}$




$\;\;\;\;{\bar{P}}_{k|k}^{\left(j\right)}=\frac{1}{2\pi c}\eta_{k}^{\left(j-1\right)}\left(P_{k|k}+\left({\hat{x}}_{k|k}-{\bar{x}}_{k|k}\right){\left({\hat{x}}_{k|k}-{\bar{x}}_{k|k}\right)}^{T}\right)$




$\;\;\;\;\;\;\;+\frac{1}{c}\left(1-\eta_{k}^{\left(j-1\right)}\right)f\left(\theta_{k}|z_{1:k-1}\right)\left(P_{k|k-1}+\left({\hat{x}}_{k|k-1}-{\bar{x}}_{k|k}^{\left(j\right)}\right){\left({\hat{x}}_{k|k-1}-{\bar{x}}_{k|k}^{\left(j\right)}\right)}^{T}\right)$


  where $c=\frac{1}{2\pi }\eta_{k}^{\left(j-1\right)}+f\left(\theta_{k}\right)\left(1-\eta_{k}^{\left(j-1\right)}\right)$ is a normalization term, and $f(\theta_{k}|z_{1:k-1})$ can be obtained using (A6).
• **Update parameters**:


$\;\;\;\;ln\left(\frac{\eta_{k}^{\left(j\right)}}{1-\eta_{k}^{\left(j\right)}}\right)=\psi \left(\alpha_{1,k}^{\left(j-1\right)}\right)-\psi \left(\alpha_{2,k}^{\left(j-1\right)}\right)+ln\left(1/2\pi \right)-lnf\left(\theta_{k}|{\bar{x}}_{k|k}^{\left(j\right)}\right)$




$\;\;\;\;\alpha_{1,k}^{\left(j\right)}=\alpha_{1,k}^{\left(j-1\right)}+\eta_{k}^{\left(j\right)}$




$\;\;\;\;\alpha_{2,k}^{\left(j\right)}=\alpha_{2,k}^{\left(j-1\right)}-\eta_{k}^{\left(j\right)}+1$


• **End for and set **${\bar{x}}_{k|k}={\bar{x}}_{k|k}^{\left(N\right)}$, ${\bar{P}}_{k|k}={\bar{P}}_{k|k}^{\left(N\right)}$, $\eta_{k}=\eta_{k}^{\left(N\right)}$, $\alpha_{1,k}=\alpha_{1,k}^{\left(N\right)}$, $\alpha_{2,k}=\alpha_{2,k}^{\left(N\right)}$.


## 5. Simulation Results

To evaluate the performance of the VB-SRF algorithm, two scenarios which are almost the same with these in [12,13] are utilized. The differences lie in the clutter probability in scenario 1 and the sensor tracks in scenario 2 which were not detailed in [12]. The two scenarios are very representative. In scenario 1, a maneuvering sensor is used in order to satisfy the condition of observability in bearings-only tracking. In scenario 2, multiple distributed sensors with large noise variance are utilized, which make the problem more challenging. The tracking performance of the VB-SRF algorithm was compared with the SRF algorithm, the MEFPDA-SCKF algorithm and PDA-SCKF algorithm in terms of track loss, track accuracy and computation complexity. The track loss is declared when the track error is large enough that making the filter diverge. Root mean square (RMS) error is used to show the track accuracy. In addition, the computation complexity is reflected by the computation time of each filter. The simulation codes can be downloaded through Github [20].

### 5.1. Scenario 1

In scenario 1, a target moves along a horizontal track, with zero vertical displacement, according to a white noise acceleration model. The state of the target is represented as $x_{k}=\left[x_{1,k},{\dot{x}}_{1,k}\right]$, where $x_{1,k}$ and ${\dot{x}}_{1,k}$ are the horizontal distance and velocity at time *k*, respectively. The observer platform follows an approximately parallel track at a constant average speed. The horizontal and vertical displacements of the platform $x_{k}^{p}={\left[x_{1,k}^{p},x_{2,k}^{p}\right]}^{T}$ are governed by the equations:

(46)x1,kp=4k+x˜1,kp(47)x2,kp=20+x˜2,kp

in which ${\tilde{x}}_{1,k}^{p}$ and ${\tilde{x}}_{2,k}^{p}$ are zero mean Gaussian white noise processes, both with variance q=1.

**Figure 1:**
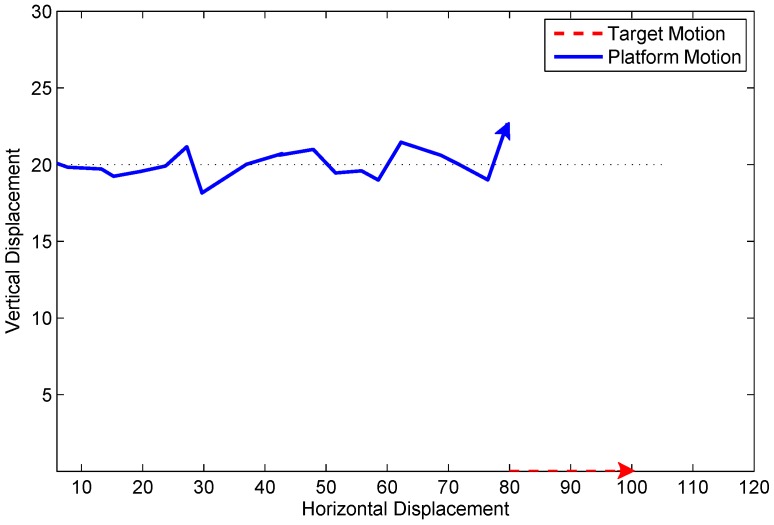
Target-observer geometry in Scenario 1.

**Figure 2:**
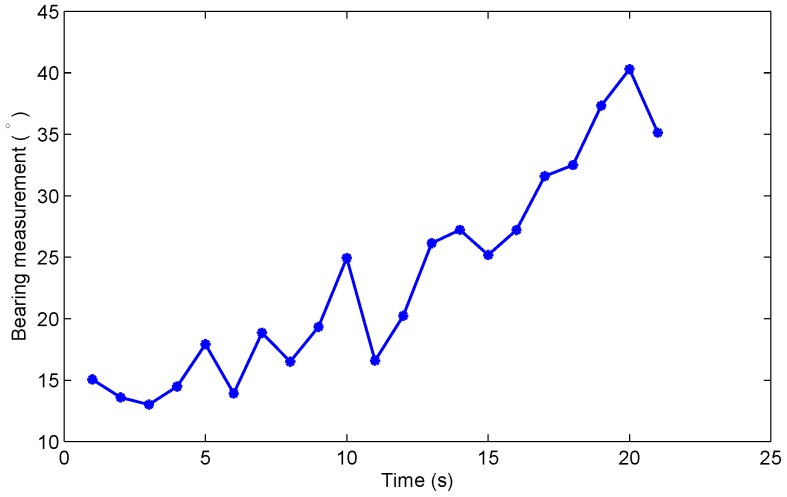
The measurement of the target in Scenario 1.

The measurement is the angle (in radians) of the line-of-sight of this target from the platform. The sensor noise is Gaussian white noise with variance $\sigma^{2}={\left(0.05\right)}^{2}{\mathrm{rad}}^{2}=2.86^{2}\deg^{2}$. The true clutter probability is 0.8. Other parameters are detailed in [13]. The configuration of the observer platform and target is illustrated in Figure 1. The bearing measurement of the target is presented in Figure 2. It can be seen that there is no abrupt change.

**Figure 3:**
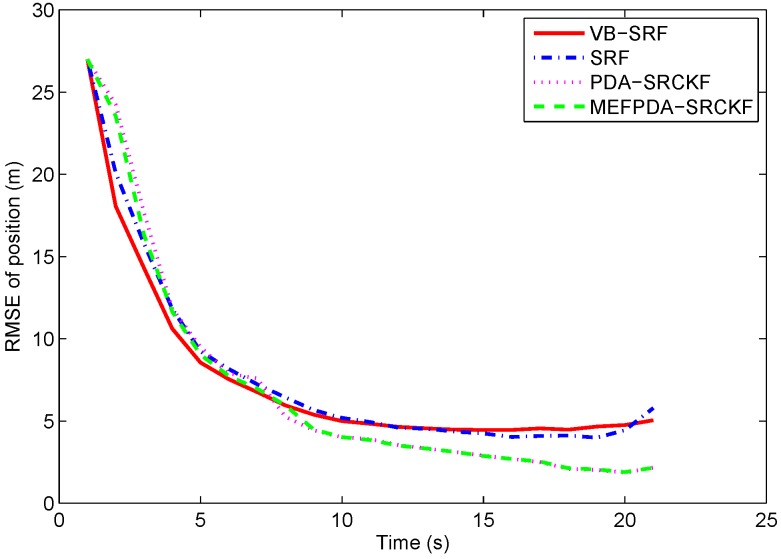
RMS target position errors with correct clutter probability in Scenario 1.

The clutter probability used in SRF is set as $p_{c}=0.8$, which is the same with the true clutter probability. The initial values of VB-SRF parameters are $\eta_{0}=0.8$, $\alpha_{1,0}=2$, $\alpha_{2,0}=10$. The clutter density λ is calculated using $-kln(1-p_{c})/2\pi$ in MEFPDA-SCKF and PDA-SCKF. Figure 3 presents the RMS target position errors of the four filters using 1000 Monte Carlo runs. It can be seen that SRF and VB-SRF have comparable performance under the correct clutter probability. MEFPDA-SCKF and PDA-SCKF have a little better tracking accuracy than SRF and VB-SRF.

In challenging scenarios, the clutter probability maybe unknown or time varying. The pre-set parameters are probably inaccurate. So here we set mistuned clutter probabilities for the four filters: (1) $p_{c}=\eta_{0}=0.7$; (2) $p_{c}=\eta_{0}=0.5$; (3) $p_{c}=\eta_{0}=0.3$. The percentages of track losses in 1000 Monte Carlo runs are given in Table 1. Clearly, the VB-SRF algorithm outperforms the SRF algorithm due to fewer track losses. Moreover the proportion of track losses increases as the mistuning aggravates. Especially, there are 2.9% tracks are lost for SRF while only 0.1% tracks are lost for VB-SRF in the worst case. In addition, we can see that MEFPDA-SCKF and PDA-SCKF have no track loss in all the three mistuned cases.

**Figure 4:**
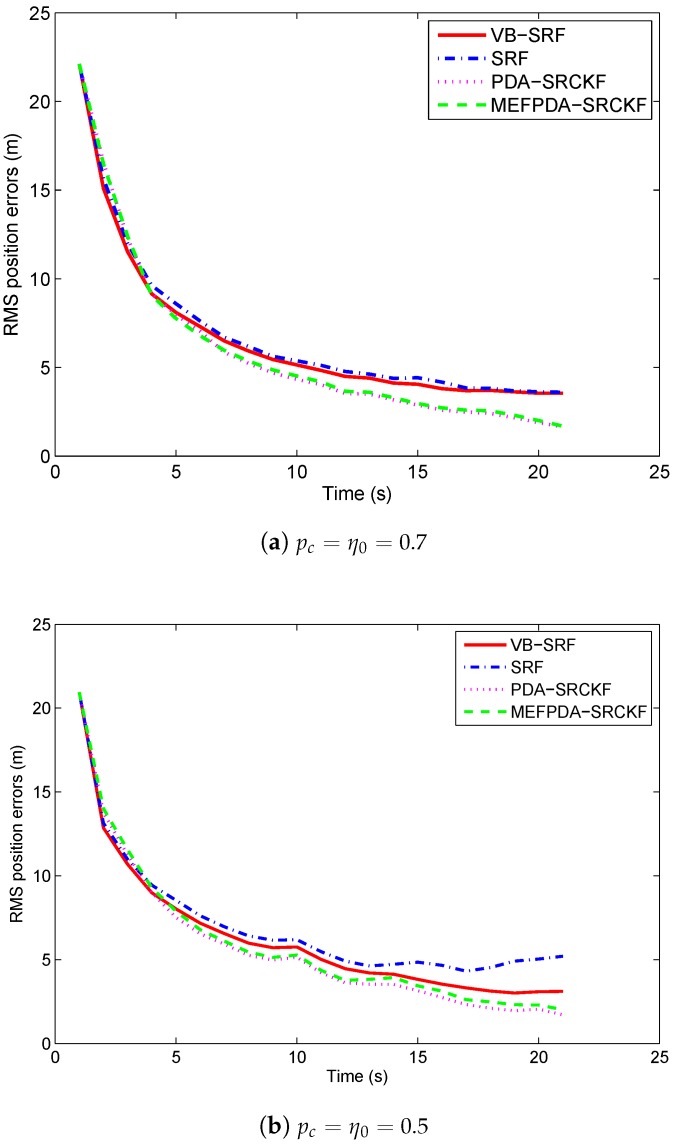

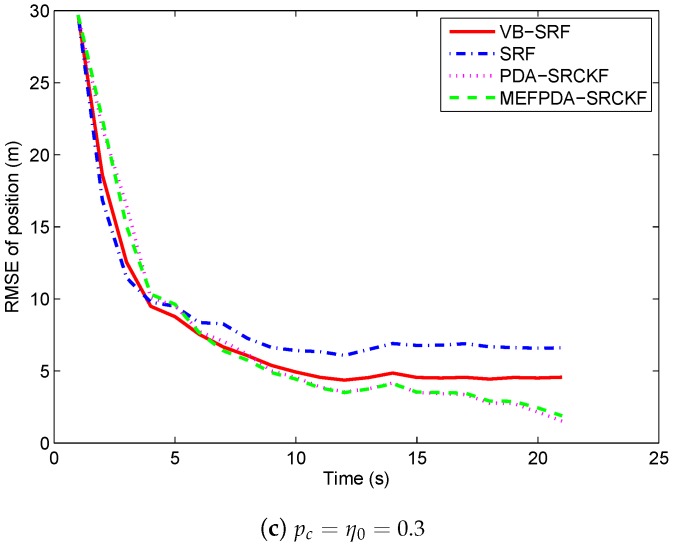
RMS target position errors with different mistuned clutter probabilities in Scenario 1.

The RMS position errors of the four filters with different mistuned clutter probabilities are shown in Figure 4. We only consider the runs without track loss. From the figure, we can see that the tracking accuracy of VB-SRF is slightly better than SRF when $\eta_{0}\geq 0.5$. However, the performance difference is not obvious. When $p_{c}=\eta_{0}=0.3$, VB-SRF exhibits distinct superiority over SRF. It implies that VB-SRF is more robust than SRF. Meanwhile, MEFPDA-SCKF and PDA-SCKF show better tracking accuracy than SRF and VB-SRF under all the three mistuned cases. They are almost not affected by the mistuning. It shows MEFPDA-SCKF and PDA-SCKF are more accurate and robust than SRF and VB-SRF under this simple scenario.

Table 2 shows the computation time of the four filters with 100 Monte Carlo runs. It is clear that VB-SRF has the maximum time of computation, which is about 2 times of that of SRF. The computation time of MEFPDA-SCKF and PDA-SCKF are comparable and both smaller than SRF and VB-SRF.

On the whole, the VB-SRF algorithm outperforms the SRF in terms of track continuity, track accuracy and robustness especially in severely mismatched scenarios but with higher computation complexity. MEFPDA-SCKF and PDA-SCKF perform better than SRF and VB-SRF in all aspects. It illustrates that the PDA-SCKF-based strategy has superiority over the SRF-based strategy in handing the clutters in simple scenarios.

### 5.2. Scenario 2

For scenario 2, the aim is to track a single target from several drifting sonobuoys. A monitoring aircraft estimates the positions of the drifting sonobuoys by observing the direction of arrival of sensor transmissions. The sonobuoys track the position of the target by means of noisy bearings measurements.

The state is 12-dimensional:

(48) $$x_{k}={\left[x_{0,k},{\dot{x}}_{0,k},y_{0,k},{\dot{y}}_{0,k},x_{1,k},y_{1,k},x_{2,k},y_{2,k},x_{3,k},y_{3,k},u_{1,k},u_{1,k}\right]}^{T}$$

the first four components represent the (x,y) coordinates of the position and velocity of the target, the next six, the coordinates of the positions of the three sonobuoys, and the last two, those of the drift current effecting all three sonobuoys.

**Figure 5:**
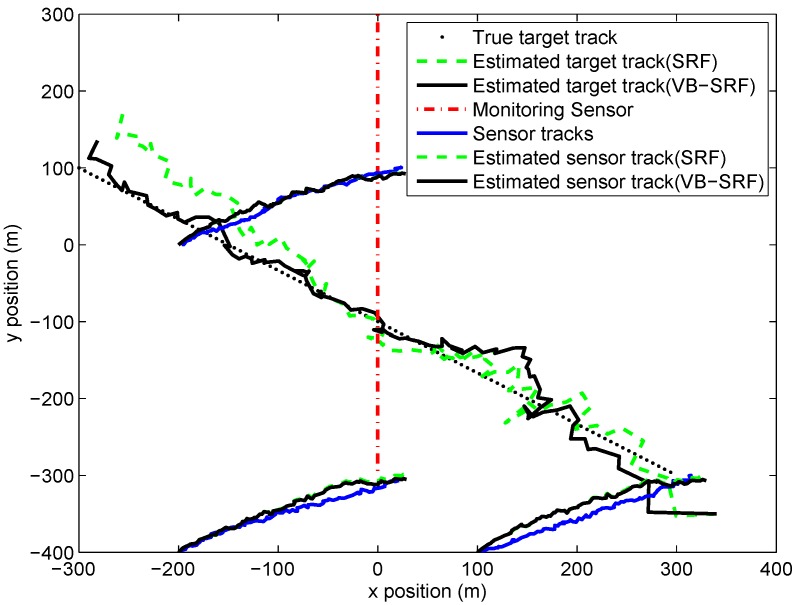
Typical tracks of target, drifting sonobuoy sensors, together with the estimated tracks.

Six simultaneous measurements are made at each time step. Three of these are measurements of the bearing angles of the sonobuoys from the monitoring platform and they are uncluttered. Three are the bearing angles of the target from the sonobuoys, which are subject to clutter. The standard deviation of monitoring sensor bearing noise and sonobuoy sensor bearing noise are 0.8° and 16°, respectively. The true probability of clutter is set as 0.667. In addition, the bearing of the clutter is uniformly distributed over [−π,π]. Other simulation parameters can be referred to [12]. 200 Monte Carlo runs are performed to evaluate the performance of the proposed filter. Figure 5 shows the behaviour of the estimates of target and sonobuoy positions provided by both the SRF and VB-SRF, for a typical simulation.

**Figure 6:**
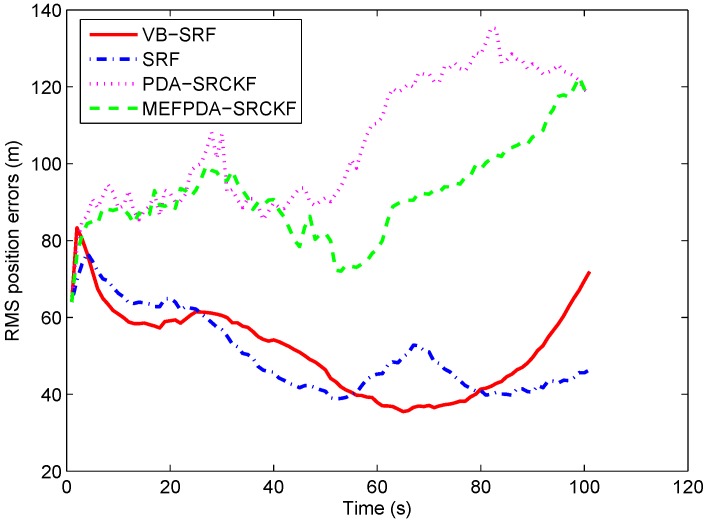
RMS target position errors with correct clutter probability in Scenario 2.

**Figure 7:**
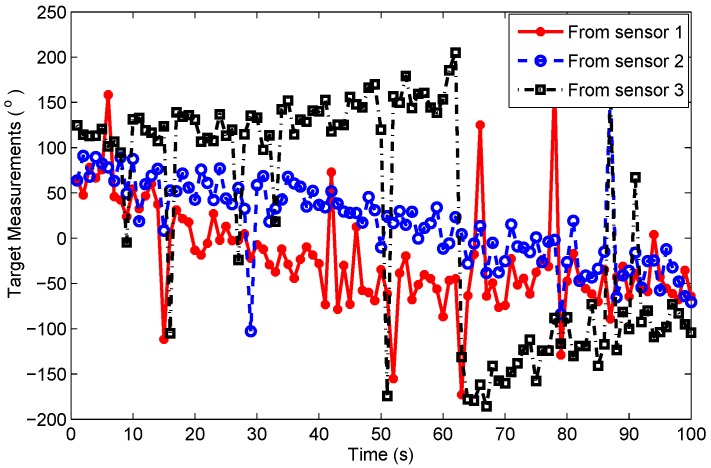
The target measurements from three sonobuoy sensors in Scenario 2.

The RMS target position errors of the four filters with correct clutter probability assumption are given in Figure 6. Compared with scenario 1, the differences between the four filters are more dramatic in scenario 2. This is probably because scenario 2 is more complex in which multiple sensors are used to observe the target and give abruptly changing and severely noise-corrupted bearing measurements of the target, shown in Figure 7. Thus, even a minor change in filtering strategy could result in large variations in performance. Meanwhile, seen from Figure 7, an abrupt change (almost from +180° to −180°) occurs in the target bearing measurement from sonobuoy sensor 3 at *k* = 62 s, which leads to several track losses shown in Table 1 and much larger position errors of MEFPDA-SCKF and PDA-SCKF. Whereas, SRF and VB-SRF are less affected by the abrupt bearing variation since the value of the projected measurement $b_{k}={\left(sin\theta_{k},cos\theta_{k}\right)}^{T}$ is invariant when there is a 360° change in bearing $\theta_{k}$. They have comparable tracking accuracy. It is hard to decide which one is better.

**Figure 8:**
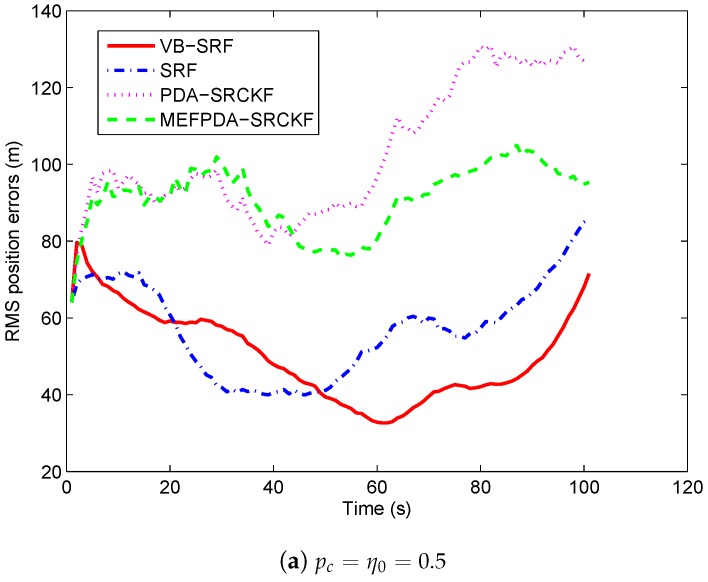

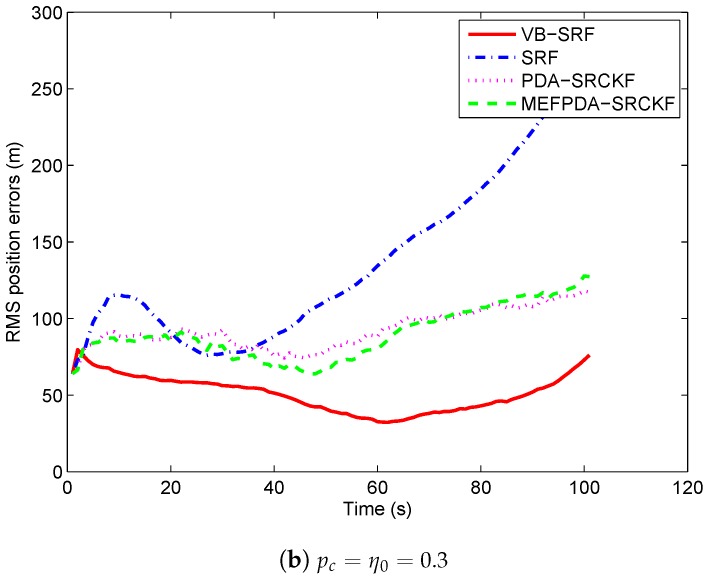
RMS target position errors with mistuned clutter probability in Scenario 2.

To compare the performance under adverse scenarios, we set mistuned clutter probabilities as: (1) $p_{c}=\eta_{0}=0.5$; (2) $p_{c}=\eta_{0}=0.3$. In case (1), there is no track loss in SRF and VB-SRF and the RMS position errors of the two filters are shown in Figure 8a. We can see that the RMS position errors of SRF are slightly increased compared with the case with no mistuning, while the RMS position errors of VB-SRF remain almost unchanged. In case (2), as shown in Table 1, 2.7% tracks are lost for SRF while no track is lost for VB-SRF. Meanwhile, as can be seen in Figure 8b, VB-SRF has much smaller RMS errors than the SRF. For MEFPDA-SCKF and PDA-SCKF, unlike with scenario 1, they have higher percentage of track losses and larger RMS position errors than VB-SRF in both mistuned cases. It shows that VB-SRF is superior to PDA-SCKF-based algorithms in more challenging scenarios. In addition, from Table 2, we can see that the computation time of VB-SRF is twice of the SRF and three times of MEFPDA-SCKF and PDA-SCKF. Overall, all these reveal that the proposed VB-SRF algorithm has significant performance superiority in severely mismatched and complex cases at the cost of a little higher computation complexity.

## 6. Conclusions

Bearings-only target tracking in the presence of clutter is a difficult problem because of the nonlinearity of the measurement model, the measurement origin uncertainty and the observability of the target. The Shifted Rayleigh filter (SRF) is shown to exhibit good performance for bearings-only target tracking in certain challenging scenarios through exploiting the essential structure of the nonlinearities in a new way. However, the clutter probability is assumed known and constant in SRF, which may not match with the truth especially in adverse scenarios. Therefore, to handle the bearings-only target tracking in clutters with uncertain clutter probability, a variational Bayesian-based adaptive shifted Rayleigh filter (VB-SRF) is proposed in this paper. By establishing a conjugate exponential model of the clutter probability and the data association indicator, the approximated posterior probability densities of the target state and the clutter parameters are iteratively calculated using the VB expectation and maximization steps. Finally, joint estimation of the target state and the clutter probability are achieved in the framework of SRF. The tracking performance of the proposed filter is compared with SRF, PDA-SCKF and MEFPDA-SCKF via two simulation examples. It shows that the proposed filter outperforms the other three filters in terms of track continuity and track accuracy with a little higher computation complexity in complex adverse scenarios. In addition, it also reveals that the proposed VB-SRF exhibits better robustness than the traditional SRF.

## Figures and Tables

**Table 1:** The percentages of track losses of four filters in two scenarios.

	Scenario 1			Scenario 2		
	$p_{c}=0.7$	$p_{c}=0.5$	$p_{c}=0.3$	$p_{c}=0.667$	$p_{c}=0.5$	$p_{c}=0.3$
VB-SRF	0	0	0.1%	0	0	0
SRF	0.9%	1.6%	2.9%	0	0	2.7%
MEFPDA-SCKF	0	0	0	13.5%	13.3%	14.2%
PDA-SCKF	0	0	0	20.1%	20.8%	22.4%

**Table 2:** Computation time of the four filters with 100 Monte Carlo runs for two scenarios.

	Scenario 1	Scenario 2
VB-SRF	0.7406 s	1.0236 s
SRF	0.3690 s	0.5779 s
MEFPDA-SCKF	0.2066 s	0.3314 s
MEFPDA-SCKF	0.2092 s	0.3128 s

## Abbreviations

The following abbreviations are used in this manuscript:

VB	Variational Bayesian
SRF	Shifted Rayleigh Filter
PDA	Probability Data Association
EKF	Extended Kalman Filter
MPEKF	Polar Coordinate EKF
PLE	Pseudo-Linear Estimator
UKF	Unscented Kalman Filter
CKF	Cubature Kalman Filter
PF	Particle Filter
MEFPDA	Maximum Entropy Fuzzy Probabilistic Data Association
SCKF	Square-root Cubature Kalman Filter
CE	Conjugate Exponential
KL	Kullback- Leibler
EM	Expectation-Maximum
RMS	Root Mean Square

## Appendix A. Derivation of *f*(*θ*_*k*_|*x*_*k*_)

Given the true state $x_{k}$, the “augmented” measurement $y_{k}$ is assumed to be $N(m_{k},Q_{w})$ variable, where $m_{k}=H_{k}x_{k}+u_{k}^{m}$. $\theta_{k}$ is the bearing of $y_{k}$.

Let $y_{k}^{\prime }=Q_{w}^{-1/2}y_{k}$, then $y_{k}^{\prime }∼N\left(Q_{w}^{-1/2}m_{k},I\right)$. Its bearing has a density about its mean of the form $\alpha_{\sigma }\left(\theta \right)$. More precisely, let

(A1) $$g_{k}\left(\theta \right)={\left[\left(sin\theta ,cos\theta \right)Q_{w}^{-1}{\left(sin\theta ,cos\theta \right)}^{T}\right]}^{-1/2}$$

and define

(A2) $$h_{k}\left(\theta \right)=arctan\left(\frac{a_{11}sin\theta +a_{12}cos\theta }{a_{21}sin\theta +a_{22}cos\theta }\right)$$

where $\left[a_{ij}\right]=Q_{w}^{-1/2}$.

Actually, $g_{k}\left(\theta \right)$ and $h_{k}\left(\theta \right)$ are the reciprocal length and bearing of the transformed unit vector $\frac{Q_{w}^{-1/2}y_{k}}{\left|\right|y_{k}\left|\right|}$. The bearing $h_{k}\left(\theta_{k}\right)$ then has $\alpha_{g_{k}\left(\theta_{k}^{m}\right)}\left(h_{k}\left(\theta_{k}\right)-h_{k}\left(\theta_{k}^{m}\right)\right)$ as its density, where $\theta_{k}^{m}$ is the bearing of $m_{k}$. Inserting a Jacobian term, we can obtain the likelihood of measurement $\theta_{k}$:

(A3) $$f\left(\theta_{k}|x_{k}\right)=\frac{g_{k}^{2}\left(\theta_{k}\right)}{{\left(detQ_{w}\right)}^{1/2}}\alpha_{g_{k}\left(\theta_{k}^{m}\right)}\left(h_{k}\left(\theta_{k}\right)-h_{k}\left(\theta_{k}^{m}\right)\right)$$

## Appendix B. Derivation of *f*(*θ_k_*|z_1:*k*−1_)

Given the previous measurements $y_{1:k-1}$, the “augmented” measurement $y_{k}$ is assumed to be $N({\hat{y}}_{k},S_{k})$ variable, where ${\hat{y}}_{k}=H_{k}{\hat{x}}_{k|k-1}+u_{k}^{m}$, $S_{k}=H_{k}P_{k|k-1}H_{k}^{T}+Q_{w}$.

Let $y_{k}^{\prime }=S_{k}^{-1/2}y_{k}$, then $y_{k}^{\prime }∼N\left(S_{k}^{-1/2}{\hat{y}}_{k},I\right)$. The bearing of $y_{k}^{\prime }$ has a density about its mean of the form $\alpha_{\sigma }\left(\theta \right)$.

Let

(A4) $$g_{k}^{\prime }\left(\theta \right)={\left[\left(sin\theta ,cos\theta \right)S_{k}^{-1}{\left(sin\theta ,cos\theta \right)}^{T}\right]}^{-1/2}$$

(A5) $$h_{k}^{\prime }\left(\theta \right)=arctan\left(\frac{s_{11}sin\theta +s_{12}cos\theta }{s_{21}sin\theta +s_{22}cos\theta }\right)$$

where $\left[s_{ij}\right]=S_{k}^{-1/2}$.

$g_{k}^{\prime }\left(\theta \right)$ and $h_{k}^{\prime }\left(\theta \right)$ are the reciprocal length and bearing of the transformed unit vector $\frac{S_{k}^{-1/2}y_{k}}{\left|\right|y_{k}\left|\right|}$. The bearing $h_{k}^{\prime }\left(\theta_{k}\right)$ then has $\alpha_{g_{k}^{\prime }\left({\hat{\theta }}_{k}\right)}\left(h_{k}^{\prime }\left(\theta_{k}\right)-h_{k}^{\prime }\left({\hat{\theta }}_{k}\right)\right)$ as its density, where ${\hat{\theta }}_{k}$ is the bearing of ${\hat{y}}_{k}$. Inserting a Jacobian term, we can obtain the probability density function of measurement $\theta_{k}$ given previous measurements $z_{1:k-1}$:

(A6) $$f\left(\theta_{k}|z_{1:k-1}\right)=\frac{g_{k}^{\prime 2}\left(\theta_{k}\right)}{{\left(detS_{k}\right)}^{1/2}}\alpha_{g_{k}^{\prime }\left({\hat{\theta }}_{k}\right)}\left(h_{k}^{\prime }\left(\theta_{k}\right)-h_{k}\left({\hat{\theta }}_{k}\right)\right)$$
